# Neuroprem: the Neuro-developmental outcome of very low birth weight infants in an Italian region

**DOI:** 10.1186/s13052-020-0787-7

**Published:** 2020-02-22

**Authors:** Licia Lugli, Marisa Pugliese, Carlotta Plessi, Alberto Berardi, Isotta Guidotti, Gina Ancora, Sara Grandi, Giancarlo Gargano, Silvia Braibanti, Fabrizio Sandri, Silvia Soffritti, Elisa Ballardini, Vittoria Arena, Marcello Stella, Serafina Perrone, Sabrina Moretti, Vittoria Rizzo, Fabrizio Ferrari, Neuroprem Working Group, Odoardo Picciolini, Roberto Bellù, Daniela Turoli, Luigi Tommaso Corvaglia, Gianpaolo Garani, Vittoria Paoletti, Giacomo Biasucci, Augusto Biasini, Belinda Benenati, Paolo Stagi, Cristina Magnani, Sara Dallaglio, Elisa DellaCasa Muttini, Maria Federica Roversi, Luca Bedetti, Laura Lucaccioni, Natascia Bertoncelli, Alessandra Boncompagni

**Affiliations:** 1 Neonatal Intensive Care Unit, University Hospital of Modena, Via del Pozzo 71, 41100 Modena, Italy; 2 Neonatal Intensive Care Unit, Infermi Hospital of Rimini, Rimini, Italy; 3 Neonatal Intensive Care Unit, Santa Maria Nuova Hospital of Reggio Emilia, Reggio Emilia, Italy; 4 Neonatal Intensive Care Unit, Maggiore Hospital of Bologna, Bologna, Italy; 5 Neonatal Intensive Care Unit, Sant’Anna Hospital of Ferrara, Sant’Anna, Italy; 6 Neonatal Intensive Care Unit, Bufalini Hospital of Cesena, Cesena, Italy; 7 Neonatal Intensive Care Unit, University Hospital of Parma, Parma, Italy

**Keywords:** Neuro-developmental outcome, Very low birth weight preterm, Italy

## Abstract

**Introduction:**

The survival of preterm babies has increased worldwide, but the risk of neuro-developmental disabilities remains high, which is of concern to both the public and professionals. The early identification of children at risk of neuro-developmental disabilities may increase access to intervention, potentially influencing the outcome.

**Aims:**

Neuroprem is an area-based prospective cohort study on the neuro-developmental outcome of very low birth weight (VLBW) infants that aims to define severe functional disability at 2 years of age.

**Methods:**

Surviving VLBW infants from an Italian network of 7 neonatal intensive care units (NICUs) were assessed for 24 months through the Griffiths Mental Developmental Scales (GMDS-R) or the Bayley Scales of Infant and Toddler Development (BSDI III) and neuro-functional evaluation according to the International Classification of Disability and Health (ICF-CY). The primary outcome measure was severe functional disability at 2 years of age, defined as cerebral palsy, a BSDI III cognitive composite score < 2 standard deviation (SD) or a GMDS-R global quotients score < 2 SD, bilateral blindness or deafness.

**Results:**

Among 211 surviving VLBW infants, 153 completed follow-up at 24 months (72.5%). Thirteen patients (8.5%) developed a severe functional disability, of whom 7 presented with cerebral palsy (overall rate of 4.5%). Patients with cerebral palsy were all classified with ICF-CY scores of 3 or 4. BSDI III composite scores and GMDS-R subscales were significantly correlated with ICF-CY scores (*p* < 0.01).

**Conclusion:**

Neuroprem represents an Italian network of NICUs aiming to work together to ensure preterm neuro-developmental assessment. This study updates information on VLBW outcomes in an Italian region, showing a rate of cerebral palsy and major developmental disabilities in line with or even lower than those of similar international studies. Therefore, Neuroprem provides encouraging data on VLBW neurological outcomes and supports the implementation of a preterm follow-up programme from a national network perspective.

## Introduction

The survival of preterm babies has increased worldwide, with a concomitant decrease in severe neonatal morbidity. However, the risk of neuro-developmental and behavioural disabilities remains high in children and adults who are born preterm. The high rates of neurological and developmental problems reported in survivors are of concern to both the public and professionals. Among extremely preterm babies (22–26 weeks gestation), reported survival without neuro-developmental impairment at 2 years is variable, from 20 to 42% [1–3]. In absolute numbers, infants born very and moderately preterm represent a larger proportion of preterm births, accounting for more children with motor, cognitive, or behavioural deficits and learning disabilities [4–6]. The early identification of children at risk of neuro-developmental disabilities may increase access to intervention, potentially improving the outcome [7]. The Bayley Scales of Infant and Toddler Development and the Griffith Mental Developmental Scale are used for neuro-developmental evaluation between 18 and 30 months, depending on the local follow-up protocols [8–10]. Both are costly, time consuming, and require trained staff. Alternative tools that can effectively screen for developmental delay in a uniform way are therefore needed. Neuro-functional evaluation according to the International Classification of Disability and Health is accepted by the World Health Organization (WHO) as a valid developmental screening tool and is becoming increasingly popular to assess development [11]. The International Classification of Functioning, Disability, and Health – Children and Youth version (ICF-CY) (World Health Organization, 2007) endorses the biopsychosocial model and comprehensively addresses disability by linking neuro-developmental outcomes to social and environmental factors [11]. Different studies have shown the value of ICF-CY-based datasets to compare functioning and disability data in children of different ages [12, 13]. Moreover, the ICF-CY-based approach has been implemented successfully in routine follow-up programmes for preterm infants [14, 15]. It is easy to administer and interpret; it has a short completion time and it can decrease data heterogeneity. It enables clinicians to focus on those children suspected of having developmental delay and hence needing further developmental assessment or intervention.

Updated data on the neuro-development of very low birth weight (VLBW) infants are scant, and national networks on preterm neurological outcomes are still lacking in Italy.

This is an area-based prospective cohort study on the neuro-developmental outcome of preterm infants that aims to define severe functional disability at 2 years of corrected age among VLBW infants in an Italian region. For this purpose, different neuro-developmental evaluation instruments (the Bayley Scales of Infant and Toddler Development, BSDI III; the Griffith Mental Developmental Scale, GMDS-R; and the ICF-CY) were used and compared.

## Methods

Neuroprem represents an Italian network of NICUs aiming to work together to ensure preterm neuro-developmental assessment. In 1999, the NICUs of an Italian region (Emilia Romagna) joined the Vermont Oxford Network Database [16] and in 2015–2016 set up this prospective cohort study on the neuro-developmental outcome of VLBW infants. Before starting patient enrolment, NICUs participated in seminars and meetings to define and share the study protocol. A common data collection form was created, including perinatal and neuro-developmental follow-up data. Anonymized data were collected through a web platform. For the purpose of this study, the cohort of VLBW infants born in 2016 was enrolled, and their neuro-developmental data at 24 months, corrected for prematurity, were collected. Surviving infants were assessed with neurological examination according to the Amiel-Tison neurological assessment [17] and either with the Griffiths Mental Developmental Scales (GMDS-R, 1996) (18) or the Bayley Scales of Infant and Toddler Development (BSDI III, 2006) [18], depending on the local protocols.

GMDS-R (0–2 years) provides a General Development Quotient (GQ) of infants’ abilities with a mean of 100.5 and an SD of 11.8 and five subscale quotients (Locomotor, Eye & Hand Coordination, Personal & Social, Hearing & Language, and Cognitive Performance) [19]. The BSDI III provides standardized composite scores for each of the assessed domains (cognitive, fine and gross motor, receptive and expressive language, and adaptive), with a mean of 100 and an SD of 15 [18]. For both the GMDS-R and BSID-III, the cut-off abnormality was 2 standard deviations (SD) below the normative mean. BSDI III or GMDS-R results below 2 SD were compared among 3 groups of different gestational ages.

Enrolled patients were also assessed with neuro-functional evaluation according to the International Classification of Disability and Health (ICF-CY) [11]. Neuro-functional clinical evaluation was performed for cognitive, linguistic, motor, and adaptive function, and then a global score ICF-CY was assigned (Appendix).

The rate and type of cerebral palsy were evaluated [20].

The primary outcome measure was severe functional disability at 2 years of age, corrected for prematurity. Severe functional disability was defined as follows: cerebral palsy, a BSDI III cognitive composite score < 2 SD or a GMDS-R GQ < 2 SD, bilateral blindness (visual acuity < 6/60 in better eye), or bilateral deafness (requiring bilateral hearing aids or unilateral/bilateral cochlear implants).

The correlation between ICF-CY, BSDI III or GMDS-R and cerebral palsy was evaluated.

The study was approved by the Ethical Committee. For every enrolled patient, written consent was signed by the parents.

Statistical analyses were performed using Stata Direct Statistical Software version 13 (StataCorp LP, USA). Continuous variables were described using means and SD, while categorical variables were described using frequencies. Groups were compared by χ^2^ analyses for categorical variables and by analysis of variance for continuous data. Agreement between different tests was evaluated with kappa coefficient. A *p* value < 0.05 was considered statistically significant.

## Results

A total of 228 VLBW infants were enrolled from 7 NICUs of the Emilia Romagna region (all tertiary referral centers). Seventeen died (7.4%). Among 211 survivors, 153 patients completed follow-up at 24 months (72.5%). They had gestational ages between 23 and 33 weeks (mean gestational age 29.1 ± 2.8; CI 28.6–29.5) and birth weights between 495 g and 1500 g (mean birth weight 1113.9 g ± 283.5; CI 1068.7–1159.2). No differences in gestational age, birth weight, gender, ethnicity, or parental education were found between patients who completed follow-up and patients who dropped out. These 153 patients included in the research received mechanical ventilation for a mean duration of 4.4 ± 11.4 days, non invasive ventilation for 19.0 ± 23.2 days and oxygen supplementation for 25.6 ± 33.8 days. Mean hospital stay was 54.3 ± 31.2 days and mean body weight at discharge from hospital was 2231.7 g ± 518.4. After discharge 31/153 (20.3%) infants were included in a program of neuro-motor rehabilitation.

Among 153 patients completing follow-up, 86 (56.2%) were evaluated with the BDS-III and 67 (43.8%) with the GMDS-R. Table 1 shows the BSDI III and GMDS-R results among the 3 different gestational age groups. Cognitive function evaluated with the ICF-Y score was significantly worse in the lower gestational age group, while motor, language and adaptive function were not significantly different among the 3 groups (Table 2). The rate of patients classified with an ICF-CY score ≥ 2 was significantly higher in the lower gestational age group: 14/54 (25.9%) in preterm infants with ≤28 weeks gestational age; 9/58 (15.5%) in patients with 29–30 weeks gestational age; and 2/41 (4.9%) in patients with ≥31 weeks gestational age (comparison among 3 groups, *p* = 0.0224).

**Table 1:** BSDI-III or GMDS-R evaluation and comparison among different gestational age groups

	All Patients (153)	GA ≤ 28 weeks (54)	GA 29–30 weeks (58)	GA ≥ 31 weeks (41)	*P*
Patients evaluated with GMDS-R (%)	67/153 (438)	20/54 (37)	26/58 (44.8)	21/48 (51.2)	–
GMDS-R performance subquotient < 2SD	6/67 (8.9)	3/20 (15)	3/26 (11.5)	0/21 (0)	0.2045
GMDS-R locomotor subquotient < 2SD	2/67 (3)	1/20 (5)	0/26 (0)	1/21 (4.8)	0.5196
GMDS-R hearing and language subquotient < 2SD	5/67 (7.4)	3/20 (15)	2/26 (7.7)	0/21 (0)	0.1882
GMDS-R personal & social subquotient < 2SD	1/67 (15)	1/20 (5)	0/26 (0)	0/21 (0)	0.3034
GMDS-R Eye & Hand Coordination subquotient < 2SD	5/67 (7.5)	3/20 (15)	2/26 (7.7)	0/21 (0)	0.1381
GMDS-R global quotient < 2SD	6/67 (8.9)	3/20 (15)	3/26 (11,5)	0/21 (0)	0.2045
Patients evaluated with BSDI-III (%)	86/153 (56.2)	34/54 (63)	32/58 (55.2)	20/41 (48.8)	–
BSDI-III Cognitive composite score < 2SD	3/86 (3.5)	3/34 (8.8)	0/32 (0)	0/20 (0)	0.0928
BSDI-III Motor composite score < 2SD	4/86 (4.6)	3/34 (8.8)	0/32 (0)	1/20 (5)	0.2344
BSDI-III Language composite score < 2SD	8/86 (9.3)	6/34 (17.6)	1/32 (3.1)	1/20 (5)	0.0957
BSDI-III Adaptive composite score < 2SD	8/86 (9.3)	5/34 (14.7)	2/32 (6.2)	1/20 (5)	0.3737

*GA* gestational age

**Table 2:** Neuro-functional clinical evaluation (ICF-CY) and comparison among different gestational age groups

	All patients (%)	GA ≤ 28 weeks (%)	GA 29–30 weeks (%)	GE ≥ 31 weeks (%)	*P*
Number (%)	153 (100)	54 (35.2)	58 (38)	41 (26.8)	–
Cognitive function					
1	128 (83.7)	39 (72.2)	50 (86.2)	39 (95.1)	0.0414
2	18 (11.6)	11 (20.4)	5 (8.6)	2 (4.9)	
3	7 (4.6)	4 (7.4)	3 (5.2)	0 (0)	
Motor function					
1	132 (6.3)	43 (79.6)	52 (89.7)	37 (90.2)	0.3917
2	14 (9.1)	7 (13)	5 (8.6)	2 (4.8)	
3	7 (4.6)	4 (7.4)	1 (1.7)	2 (4.8)	
Language function					
1	110 (71.9)	36 (66.7)	43 (74.1)	31 (75.6)	0.166
2	34 (22.2)	13 (24)	11 (19)	10 (24.3)	
3	9 (5.9)	5 (9.3)	4 (6.9)	0 (0)	
Adaptive function					
1	138 (90.1)	47 (87.1)	52 (89.7)	39 (95.2)	0.6328
2	9 (5.9)	4 (7.4)	4 (6.9)	1 (2.4)	
3	5 (3.3)	3 (5.5)	1 (1.7)	1 (2.4)	
4	1 (0.7)	0 (0)	1 (1.7)	0 (0)	
ICF-CY					
0	99 (64.7)	30 (55.6)	40 (69)	29 (70.7)	0.0910
1	29 (18.9)	10 (18.5)	9 (15.5)	10 (24.4)	
2	18 (11.8)	10 (18.5)	8 (13.8)	0 (0)	
3	5 (3.3)	2 (3.7)	1 (1.7)	2 (4.9)	
4	2 (1.3)	2 (3.7)	0 (0)	0 (0)	

*GA* gestational age

BSDI III composite scores and GMDS-R subscales were significantly correlated with ICF-CY cognitive, motor, language, and adaptive function (*p* < 0.01). The agreement between BSDI III composite scores and ICF-CY was 95.3% (weighted Kappa = 0.84) for cognitive domain and 95.3% (weighted Kappa = 0.86) for motor domain. The agreement between GMDS-R were and ICF-CY was 88.1% (weighted Kappa = 0.76) for cognitive performance and 92.5% (weighted Kappa = 0.75) for locomotor subscales.

Thirteen (8.5%) patients were classified with severe functional disability: 6 showed a < 2 SD GQ score and 7 had cerebral palsy (cerebral palsy overall rate of 4.5%) (Table 3). None of the patients had severe vision deficits or deafness. Among patients with cerebral palsy, three had tetraplegia, three had diplegia and one had hemiplegia. Four cases of cerebral palsy occurred in patients with ≤28 weeks gestational age (7.4% rate), 1 case of cerebral palsy occurred among patients with 29–30 weeks gestational age (1.7% rate), and 2 cases of cerebral palsy occurred among patients with ≥31 weeks gestational age (4.8%), which was not significantly different among groups.

**Table 3:** Patients with severe functional disabilities

Case	Gestational age (weeks)	Weight (gr)	Severe functional disability	ICF-CY
1	30	1080	Tetraplegia	3
2	34	1480	Diplegia	3
3	23	550	Tetraplegia, BSDI-III cognitive composite score < 2 DS	4
4	31	1290	Diplegia	3
5	27	730	GMDS-R global quotient < 2 DS	2
6	29	1175	GMDS-R global quotient < 2 DS	2
7	28	1100	GMDS-R global quotient < 2 DS	2
8	29	930	GMDS-R global quotient <2 DS	2
9	30	1130	GMDS-R global quotient < 2 DS	2
10	28	700	GMDS-R global quotient < 2 DS	2
11	26	1050	Tetraplegia, BSDI-III cognitive composite score < 2 DS	4
12	28	1248	Hemiplegia, BSDI-III cognitive composite score < 2 DS	3
13	24	690	Diplegia	3

Among 13 patients with severe functional disability, 6 children were classified with an ICF-CY score of 2 (46.1%), 5 with an ICF-CY score of 3 (38.5%) and 2 with an ICF-CY score of 4 (15.4%). Patients with cerebral palsy were all classified with ICF-CY scores of 3 or 4 (Table 3).

There was no significant correlation between invasive/non-invasive ventilation, oxygen supplementation or hospital stay duration and outcome.

## Discussion

This is an Italian area-based study on the neuro-developmental outcome of VLBW preterm infants. In this cohort prospective study, the rate of severe functional disability was 8.5%, and the rate of cerebral palsy was 4.5%. Other follow-up studies revealed a higher prevalence of developmental disabilities in very preterm neonates, as cerebral palsy was previously reported in 10–15% of cases [21–25]. It could be claimed that our encouraging results depend on a selection bias due to the follow-up dropout rate. The group of patients completing the 24-month follow-up (72.5%) and the group who was lost to follow-up were comparable for gestational age, birth weight, ethnicity and parental education. Therefore, there was no additional known risk factor for neuro-developmental disabilities in the group lost to follow-up; moreover, the follow-up dropout rate we found was similar to that reported in previous studies. The lower rate of developmental disabilities of our study probably addresses differences in the selected study population. While most of the previous studies focused on extremely or very preterm newborns, in our study, we included VLBW infants, among whom gestational age is extremely variable (from 23 to 33 weeks of gestation). VLBW preterm infants have been poorly investigated in the past, but knowledge of their neuro-developmental trajectory could lead to targeted intervention and prevention of later disabilities, as timely intervention has a positive influence on cognitive outcome in infancy [1–3, 6]. Indeed, in the Neuroprem study a considerable number of patients was included in a program of neuro-motor rehabilitation, precisely because of prematurity risk factor or because of early sign of neurological abnormalities. Probably their neuro-developmental outcome was influenced by the precocity of these interventions. On the other hand, we have not found any correlation between ventilation, oxygen supplementation or hospital stay duration and outcome, but further larger studies focusing on the effect of perinatal factors on outcome are desirable.

We have insufficient data to evaluate the impact of the etiology of the VLBW on neuro-developmental outcome, but in our study the rates of cerebral palsy did not differ significantly among gestational ages, and cerebral palsy occurred even in preterm infants with ≥31 weeks of gestation. Additionally, the BSDI III and GMDS-R results were comparable in patients with different gestational ages, but the small sample size may have contributed to defeat the statistical analysis. In contrast, cognitive function, evaluated with the ICF-CY in the whole study population, was significantly worse in the lower gestational age group, while motor, language and adaptive function were not significantly different among groups. This means that very and extremely preterm infants are at higher risk of neuro-developmental disabilities, but moderately preterm infants may also have neurological abnormalities [4, 6, 26]. Considering these results, a follow-up programme should also include moderately preterm infants and at least VLBW infants. The strength of our research is the prospective and area-based design of the study. Neuroprem created a network of Italian NICUs, adopting a definite follow-up programme for VLBW neuro-developmental outcome assessment. Such neonatal follow-up networks may contribute to guiding health policies and increase access to formal neuro-developmental evaluation and subsequent rehabilitative interventions [7, 24]. Actually, developmental delay rather than survival is recognized as the main problem in children born preterm [1–3, 6]. The early identification of children at risk of later developmental difficulties may increase access to intervention, potentially influencing the course of otherwise persistent difficulties.

The ICF-CY-based approach has been implemented in follow-up programmes for preterm infants; it is a valid neuro-developmental screening and leads to overcoming dataset heterogeneity due to the local protocol of evaluation [14, 15]. It allows clinicians to focus on those children suspected of having a developmental delay and hence needing further developmental assessment or rehabilitation. In our study, ICF-CY subscales significantly correlated with BSDI III composite scores and GMDS-R subscales and their agreement was high. Moreover, the ICF-CY permitted the recognition of all patients with cerebral palsy or severe functional disability, leading to a targeted intervention.

The current study has some limitations. First, the study population is small, and the enrolment was performed for only one year, which may not be representative of the trend over years. Second, the follow-up is limited to the first 2 years of life, and only severe functional disabilities were investigated; therefore, we did not evaluate mild neurological dysfunction and preschool age performance, which may be impaired in different neuropsychological domains even in patients without major disabilities [5, 21, 27]. Nevertheless, this study is a first attempt to highlight the status of VLBW outcomes in an Italian region. Third, developmental quotient data are heterogeneous because either the BSD-III or the GMDS-R was used, depending on the local protocol. The standardization of developmental tests among centres is desirable, but it requires staff training and is costly and time consuming. To overcome data heterogeneity coming from the BSD-III and GMDS-R, ICF-CY was adopted by all centres, and a good correlation between the ICF-CY, BSD-III and GMDS-R was achieved.

In conclusion, this study has contributed to updating information on outcomes for VLBW infants in an Italian region. Indeed, Neuroprem has started to build up an Italian network of NICUs that aim to work together to ensure VLBW neuro-developmental follow-up. Therefore, Neuroprem may represent a model for preterm follow-up programme implementation ad neuro-developmental outcome data collection.

## Conclusion

Neuroprem represents an Italian network of NICUs aiming to work together to ensure preterm neuro-developmental assessment. We report on an Italian area-based prospective cohort study on the neuro-developmental outcome of very low birth weight (VLBW) preterm infants. This study updates information on VLBW outcomes in an Italian region, showing a rate of cerebral palsy and major developmental disabilities in line with or even lower than those of similar international studies. Therefore, Neuroprem provides encouraging data on VLBW neurological outcomes and supports the implementation of a preterm follow-up programme from a national network perspective.

## Supplementary information

**Additional file 1.** Appendix A Neuro-functional clinical evaluation

## Data Availability

Data and materials are available for consultation in a web platform (REDCap).
